# Reappraising social emotions: the role of inferior frontal gyrus, temporo-parietal junction and insula in interpersonal emotion regulation

**DOI:** 10.3389/fnhum.2013.00523

**Published:** 2013-09-03

**Authors:** Alessandro Grecucci, Cinzia Giorgetta, Nicolao Bonini, Alan G. Sanfey

**Affiliations:** ^1^Department of Psychology and Cognitive Science, University of TrentoTrento, Italy; ^2^Centro Nazionale Della Ricerca, Istituto di Scienze e Tecnologie Della CognizioneTrento, Italy; ^3^Department of Economics and Management, University of TrentoTrento, Italy; ^4^Donders Institute for Brain, Cognition and Behavior and Behavioral Science Institute, Radboud UniversityNijmegen, Netherlands

**Keywords:** interpersonal emotion regulation, decision-making, social interactions, mentalizing

## Abstract

Previous studies have reported the effect of emotion regulation (ER) strategies on both individual and social decision-making, however, the effect of regulation on socially driven emotions independent of decisions is still unclear. In the present study, we investigated the neural effects of using reappraisal to both up- and down-regulate socially driven emotions. Participants played the Dictator Game (DG) in the role of recipient while undergoing fMRI, and concurrently applied the strategies of either up-regulation (reappraising the proposer's intentions as more negative), down-regulation (reappraising the proposer's intentions as less negative), as well as a baseline “look” condition. Results showed that regions responding to the implementation of reappraisal (effect of strategy, that is, “regulating regions”) were the inferior and middle frontal gyrus, temporo parietal junction and insula bilaterally. Importantly, the middle frontal gyrus activation correlated with the frequency of regulatory strategies in daily life, with the insula activation correlating with the perceived ability to reappraise the emotions elicited by the social situation. Regions regulated by reappraisal (effect of regulation, that is, “regulated regions”) were the striatum, the posterior cingulate and the insula, showing increased activation for the up-regulation and reduced activation for down-regulation, both compared to the baseline condition. When analyzing the separate effects of partners' behavior, selfish behavior produced an activation of the insula, not observed when subjects were treated altruistically. Here we show for the first time that interpersonal ER strategies can strongly affect neural responses when experiencing socially driven emotions. Clinical implications of these findings are also discussed to understand how the way we interpret others' intentions may affect the way we emotionally react.

## Introduction

Perspectives on affective neuroscience suggest that brain structures which generate emotional responses can be successfully regulated by control regions when subjects are asked to apply cognitive strategies to emotion eliciting stimuli such as unpleasant pictures (Golkar et al., 2012; Ochsner et al., 2012). Emotion regulation (ER) refers to a set of different strategies by which “individuals influence which emotions they have, when they have them, and how they experience and express these emotions” (cf. Gross, 2007). Although mechanisms of basic emotion self-regulation have been, at least in part, recently uncovered, surprisingly little empirical work exists on an important topic: the specific neurocognitive mechanisms behind interpersonal emotion regulation (IER), a particular form of ER applied to socially driven emotions.

ER can refer not only to people's capacity to manage their own emotions, but importantly can also extend to regulating emotions that result from the interaction with others (Grecucci, 2012; Grecucci et al., 2013b). Previous studies have examined the processes that individuals use to influence which emotions they generate, when they do so, and how these emotions are experienced or expressed (Gross, 1998), and therefore we know that different attentive, behavioral, emotional, or interpretative strategies can be used also at an interpersonal level (Fonagy, 2006). Of particular interest for the present paper are studies examining the use of a strategy to regulate an existing or ongoing emotional response, typically known as reappraisal. This strategy involves reinterpreting the meaning of a stimulus to change one's emotional response to it (Gross, 1998). Subjects are usually asked in this context to build an interpretation of the emotional stimulus in such a way as to increase or decrease their emotional response (respectively, up- and down-regulation), and behavioral studies have shown that reappraisal is one of the most efficient strategies for regulating negative emotional responses (Gross, 2002). However, reappraisal applied to interpersonal contexts, that is, focusing on the interpretations of others'intentions, is relatively neglected in the literature. Despite the existence of an extensive literature on emotion “self-regulation,” focused primarily on the regulation of basic emotions such as fear and disgust in relation to visual stimuli (Ochsner and Gross, 2005, for a review), research on regulation in social interactive situations (e.g., IER) is scant (e.g., Koenigsberg et al., 2011; Grecucci et al., 2013a,b; Grecucci and Sanfey, 2013).

Notably, processing socially cued emotions engages differential networks than does non-socially cued emotion (Britton et al., 2006; Harris et al., 2007; Lestou et al., 2008), thus motivating further exploration of the regulation of socially induced emotions. The interest on such “social regulation” has been explored in a recent study examining the ER of subjects while looking at pictures depicting social vs. non-social scenes (Koenigsberg et al., 2011). This study had subjects observing emotional vs. neutral pictures while applying reappraisal strategies, but the novelty of the study was in the usage of a subset of International Affective Pictures depicting scenes with social features (e.g., people in situations of loss, abuse, aggression…) instead of simple emotional pictures. Interestingly, exposure to pictures depicting social situations activated brain areas partially involved in social cognition, such as the superior and middle temporal gyri, in addition to emotional and cognitive structures similar to previous non-social studies.

However, though in this study people were asked to reappraise emotions elicited by pictures depicting social scenarios, they were not exposed to real social interactive situations. Studying the neural systems involved in the regulation of actual interpersonal situations is particularly important given the relevance of failure in regulating interpersonal responses in psychiatric disorders (Phillips et al., 2003; Ochsner and Gross, 2008; Grecucci, 2012; Grecucci et al., 2013c). Moreover, in the aforementioned study (Koenigsberg et al., 2011) a particular form of reappraisal was used, namely “distancing,” a “self-focused” strategy in which subjects view an emotional stimulus from the perspective of a detached and distant observer (Koenigsberg et al., 2011; Ochsner and Gross, 2013). This strategy may be reasonable when looking at a picture but its use may be detrimental when interacting with a real person. In contrast, in the present study we aimed to use a reappraisal strategy focused on the “intention of others,” which involves a reinterpretation of the meaning of the other person's mind, behavior and intentions. One advantage of the latter strategy is that reinterpretation can be in both more or less negative directions thus providing the opportunity to study both up- and down-regulation effects, whereas distancing is only intended to down-regulate one's emotions. This is of notable importance given that some clinical populations (e.g., paranoid and borderline personality disorders, anxiety, schizophrenia, etc.) are characterized by interpreting the intentions of others in a malevolent way, thus causing inappropriate interpersonal and emotional reactions (Grecucci et al., 2013c). Clinicians of different schools defined this process as “projective identification” (Klein, 1946; Clarkin et al., 2006) or “hypermentalizing” (Allen and Fonagy, 2006).

In a previous study we tried to fill this gap by employing research paradigms designed to explore social economic decisions, and we evaluated whether interactive emotion regulation can occur through the mechanisms involved in self-regulation of negative emotions. These studies (Grecucci et al., 2013a,b) showed that an IER strategy of *reappraising the intentions of the other player as less negative*, or mentalizing-reappraisal (a particular kind of “reappraisal”), is effective in changing both interpersonal decisions (i.e., rejection rates of unfair offers in the context of a socio-economic game) (Grecucci et al., 2013a), as well-subjective responses to emotion themselves (Grecucci et al., 2013b). The task used in one of these experiments (Grecucci et al., 2013a) was the classic Ultimatum Game, where participants played the role of responder (Guth et al., 1982). The study showed that subjects' decisions were strongly modulated by the reappraisal strategy used: less rejections of unfair offers when down-regulating their emotions and more rejections when up-regulating their emotions. The modulation was visible in an area of the brain previously involved in the aversive reactions elicited by unfair offers, namely the insula. The posterior part of the insula showed a similar pattern of activation as was shown behaviorally (less activity for down- and more for up-regulation as compared to the neutral baseline). A limitation of that study was that the task required subjects to respond to economic offers with the possibility of rejecting the bad proposals, thus leading to lesser gain for the proposer him or herself. That is, subjects could punish proposers for the bad behavior directed toward them, and indeed, one of the primary emotions subjects reported when treated unfairly was anger. Therefore, it could well be that the punishment that subjects could inflict on proposers was itself a way to show their feelings and thus modulate their own emotional states. In other words, behavioral and neural responses showing modulation according to the reappraisal strategies could have been more concerned with the decision than with the socially induced emotions themselves. To further examine, at the neural level, how purely socially induced emotions are regulated, the same subjects played another socio-economic game called the Dictator Game (DG) (Kahneman et al., 1986), once again as responders. In the DG players must passively accept socioeconomic offers, usually both fair and unfair, and therefore do not have the possibility to punish the proposers' unfair behavior and to potentially vent their anger. Importantly, in this task we can focus more on the neural activations, without the complication of having subjects involved in both making a decision and providing a motor response. In other words we have the unique opportunity to observe the neural effects of the regulation of socially elicited emotions without the involvement of other decision processes.

In terms of the neural structures involved in ER, the literature on “self” regulation typically distinguishes regions that implement the strategy (Regulating regions) and regions that are modulated by the strategy (Regulated regions). According to a recent model of the cognitive control of emotions (MCCE, Ochsner et al., 2012), the regions involved in emotion generation that can be regulated, are, in order of importance: the amygdala, with less evidence for other regions such as the ventral striatum, the ventromedial prefrontal cortex (wmPFC), and the insula. At the same time, other regions appear to act as control systems that implement the regulatory strategy. These regions are primarily the dorsolateral prefrontal cortex (dlPFC), the anterior cingulate cortex (ACC), the ventrolateral prefrontal cortex (vlPFC) and the dorsomedial prefrontal cortex (dmPFC). However, we do not know if this model can be applied to the context of IER. Social emotions rely on different mechanisms and activate different brain areas as do non-social emotions (Britton et al., 2006), and therefore IER may be of a qualitatively different nature from self-emotion regulation (Grecucci and Sanfey, 2013).

Thus, the first goal of the present study is to identify neural correlates and possible modulations of the regulation of socially induced emotions stemming from interactive situations. In particular we aim to uncover how dedicated brain areas respond to the implementation of mentalizing-reappraisal strategies (we define them: “Regulating regions”) when regulating socially induced emotions such as those elicited by selfish and altruistic behaviors during a DG. Given the particular interactive task used in the present study, we expect that brain areas more connected with building an interpretation of others' minds and intentions will be activated, specifically the temporo-parietal junction (TPJ). In recent years TPJ activation has been connected to both social perception (Allison et al., 2000; Kourtzi and Kanwisher, 2000) as well as to attributing intentions (Van Overwalle, 2009) and mental states to others, namely theory of mind (Frith and Frith, 2003). These results can extend a useful model of ER (e.g., the MCCE) by adding social—interpersonal mechanisms.

A second goal of the present study is to explore brain regions that are modulated by these strategies (“Regulated regions”). We expect social interactions to involve different neural structures as compared to those of observing “scenes of humans interacting in a negative way” (such as scenarios of aggression or mourning). A recent study found that an emotional structure involved when looking at social emotional pictures was the amygdala (Koenigsberg et al., 2011), which is likely connected with the unpleasantness of those scenarios themselves than to the interpersonal reactions. In contrast, previous studies involving fair and unfair socioeconomic behaviors have shown that the insula may be responsible for negative reactions when treated unfairly by another player in the Ultimatum game (Sanfey et al., 2003), and thus we expect that the insula will be active in the present study when subjects are treated unfairly. We will test explicitly for the emotions invoked by assessing affective reactions following the game play. Based on the two previous goals we aim to determine the neural circuitry underlying interpersonal regulatory processes. In line with previous studies we expect a network of areas working together in order to produce successful regulation of emotions elicited by social situations. This will be formally tested in a dynamic causal modeling (DCM).

Strictly related with goal one and two, the third goal of the study is to inquire what happens when we reappraise in a negative way the intentions of others. The vast majority of the previous studies focused their attention on the effect of down regulating one's emotion. However, understanding what happens when we up-regulate emotions is of critical importance. The up-regulation of the emotion is commonly observed in psychiatric patients (in the form of excessive emotional reactivity or inappropriate emotionally laden behaviors), and it has been hypothesized to be caused by failures in the way we interpret others'intentions (Allen and Fonagy, 2006; Clarkin et al., 2006). The way we interpret others' mind, indeed affects the way we emotionally respond. This is of undeniable relevance as it covers many clinical phenomena associated with negative style of thinking and its effect on interpersonal emotional reactions as visible in paranoid, borderline patients and related disorders. The paradigm used in this experiment gives us the opportunity to have subjects reappraising events in a more or less negative way, thus providing the opportunity to study both up- and down-regulation effects on the brain and on emotional perception.

Finally, a fourth goal of this study is to detect both common and different brain regions and subjective experience, associated with the experience of being treated fairly, moderately unfairly or very unfairly.

## Methods

### Participants

Twenty-one participants (11 males, mean age: 23.5 ± 3.6 years) participated in the study. Participants had normal or corrected to normal vision and had no history of psychiatric, medical or neurological illness, as verified by a semistructured interview by a physician. All participants provided written informed consent, as approved by the local ethical committee, and were paid 35 euros for participation.

### Assessment, training procedure, paradigm, and formal debriefing

The experimental procedure comprised of four phases. A general cognitive and emotional assessment (including the Emotion Regulation Questionnaire, ERQ, Gross and John, 2003), followed by training and testing in ER techniques. Then, subjects underwent scanning with fMRI while playing rounds of the Ultimatum Game and DG under conditions of ER in two separate runs intermingled by a break. Finally, there was a formal debriefing phase. Importantly, the sequence of the 4 phases was fixed having the subjects performing first the training, than the UG, followed by the DG and finally the debriefing. Participants were told they will be playing with every partner twice in two different games (UG and DG). They were also told that partners were real and that they made two independent offers (one per game) recorded before running the experiment. The two offers were randomly assigned to every player in a way to avoid carry over effects of reputation from one game to the other. In a previous paper we reported results on Ultimatum Game (Grecucci et al., 2013a), therefore in the present paper we concentrate on the results of the DG task.

**Figure 1:**
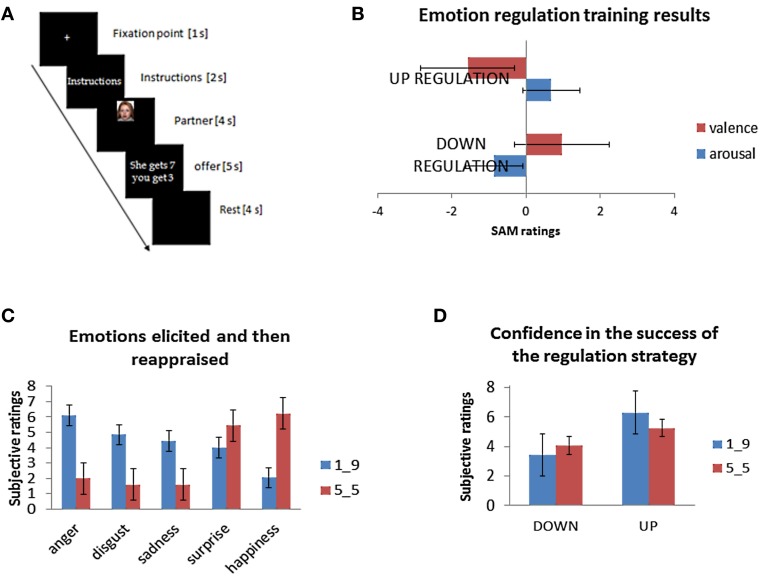
**(A)** A timeline of the events for each trial. **(B)** Emotion regulation training results before the Dictator Game ensured that subjects were able to apply the strategies. **(C)** Emotion ratings clarified the emotions primarily evoked by the task. **(D)** Subjects reported their emotions changing according to both strategies.

In line with the previous formal operationalization of mentalizing-reappraisal (see Grecucci et al., 2013b), participants were asked in the training phase to reappraise the social situation following formal instructions. “When you are required to “up-regulate” you should interpret the intentions and behavior of your partner as more negative or potentially bad (instruction: “increase”); when you are required to “down-regulate” you should interpret the intentions and behavior as less negative or potentially good (instruction: “decrease”), when you are required to “look” you should try to perceive the situation spontaneously as it is without any effort to build any particular interpretation of it.” They were given an example of a common negative situation and how it can be reinterpreted (reappraised) in such a way as to make it either more or less negative (See Grecucci et al., 2013b). To ensure subjects understood the instructions and were successfully applying the required reappraisal strategies, they were asked to reappraise while viewing pictures from the IAPS picture set (Lang et al., 1997). Eighteen unpleasant IAPS pictures were selected and divided into three subsets to be used across the reappraisal conditions (up, down, and look). After a picture was presented for 5 s, participants rated them according to valence and arousal dimensions using the Self-Assessment Manikin procedure (Lang, 1994). If the experimenter was satisfied by the reappraisal strategies used, the participant was introduced to the last part of the training, the DG. First, instructions were given on the DG (see Figure 1A for a timeline). The task instructions emphasized that the different partners in the game would play the game independently of each other, and participants were led to believe the games would be played for real with the set of partners they saw.

After the basic DG instructions, subjects were given instructions on how to apply reappraisal to DG. In the DG-training phase, each participant played three practice rounds of the DG as responders, twice in which they were asked to reappraise (according to the strategies indicated), and once in which they played without any reappraisal instruction (baseline condition). The instructions given on how to apply reappraisal strategies were as follows: “It is very important that you now try to apply the reappraising strategies learned in the IAPS-training to the situations evoked by the DG. In particular, you should try to come up with possible interpretations of the intentions and behaviors of the proposer in a way to make it more (up regulation) or less negative (down regulation). For example, when instructed to “increase” you may think the player is a selfish person (intentions) and wants to keep all the money (behavior). Whereas, when you have to “decrease,” you may think that the player has financial problems and is giving you the best offer they can.” In the “look” condition they were asked to read and emotionally respond to the offer in the most natural and spontaneous way. Participants were debriefed following these three practice trials and asked to report their strategies for each trial. After the training, participants entered the scanner and played a block of 20 rounds for each of the three regulation conditions counterbalanced across participants, for a total of 60 rounds as recipients, with each trial proposal involving a division of €10.

The set of offers received by each participant was pre-assigned. The set of 20 offers comprised of 7 fair offers (€5 to each player) and 13 unfair offers, defined as offering the participant less than half of the money. The unfair set was composed of 7 very unfair offers of €1, and of 6 mid-range values (2 offers of €2, 2 offers of €3 and 2 offers of €4). Half of the offers were made by a male partner, and half by a female partner. The order of partners and the pictures associated with each offer was completely randomized. Participants first saw a picture of the proposer on that round, followed by the offer of that player. After the offer was made, participants applied the reappraisal strategy required. To encourage participants to pay attention to the task it was emphasized that they would be paid according to the other players choice in the game (even though for local ethical reasons they were paid the same), and to make them responsive, they were required to press a button to advance to the next trial. In a post scan session participants were exposed to two samples of rounds (specifically on involving the fair 5:5 offer and one involving the unfair 1:9 offer) used during the scanning session and asked to evaluate the strength of emotions elicited (anger, sadness, disgust, surprise, and happiness) on a 9-point Likert scale. After each of these rounds they were also asked to indicate whether they felt their emotions were modulated according to the strategy when asked to apply up- and down-regulation on each of these sample trials.

### Scanning procedure

Whole brain distortion-corrected EPI with 32 axial slices (3-mm-thick, 1-mm gap) were collected at 4T (Bruker MedSpec MRI) with a T2^*^-sensitive gradient echo spiral pulse sequence (TR of 2.2 s, TE 33 ms, 75° flip angle, 64 × 64 data acquisition matrix). T2-weighted spin-echo scans were acquired for anatomical localization using the same slice prescription. Stimulus presentation and data acquisition were controlled using E-prime software. Responses were made with the index and middle fingers of the right hand using two buttons on a four button MRI-compatible response box.

### fMRI data pre-processing and general linear model analysis

Functional images were slice time corrected and motion corrected using SPM8 (Wellcome Department of Cognitive Neurology, London). For all participants, we acquired 738 volumes (246 each fMRI-run); the first 3 volumes were discarded for each run. In preprocessing of the data, the EPI volumes were spatially realigned to correct for movement artifacts (Ashburner and Friston, 2003) and motion corrected by distortions interactions (Andersson et al., 2001), and smoothed using 9-mm Gaussian kernel to account for residual intersubject differences (Worsley and Friston, 1995). For statistical analysis, we used the general linear model implemented in SPM8 as an event-related design and we modeled the onset of each category and convolved with the canonical hemodynamic response function (HRF, event duration = 0), then we estimated the effect size for each participant for each of the relevant 9 conditions (fair offers down-regulate, fair offers look, fair offers up-regulate, mid offers down-regulate, mid offers look, mid offers up-regulate, unfair offers down-regulate, unfair offers look, unfair offer up-regulate) using the general linear model. Because our main question concerned the regulation of the behavior of the partners in the DG, activation onsets were aligned with the display of the proposed monetary offer on each trial. Finally, the first-level analyses included also the parameters of the realignment (motion correction) as covariates of no interest. Next, we obtained 9 contrast images per participants, corresponding to the 9 conditions of interest. Statistical threshold were set to p-corr. = 0.05 corrected for multiple comparisons at the cluster level (cluster size estimated at p-unc. = 0.001), considering the whole brain as the volume of interest. Furthermore, region-of-interest (ROI) analyses were also carried out with the aim to provide additional information confirming the statistically valid inferences based on main effects and simple main effects off the random effects analysis. Each ROI consisted of a sphere of 8 mm of diameter centered around the peak of activation using Marsbar toolbox (Brett et al., 2002).

### DCM and Bayesian model selection

DCM (Friston et al., 2003) was used to explore experimentally induced modulations (Stephan et al., 2007) in key regions of interest to better understand the effects uncovered in the general linear model analyses. DCM models can shed light on how the neural dynamics are shaped by experimentally controlled manipulation. With DCM we aimed to test which regions were involved in the effect of ER of social interactive situations. To ensure compatibility, the choice of subject-specific coordinates was guided by group maxima as derived by the GLM analyses, and adapted to each individual by adjusting for closest maxima. Regional time series of each subject was extracted as the first eigenvariate of all activated voxels within a 8 mm radius around the maxima. BMS was based on the same GLM model of the RFX analyses described above.

## Results

### Ratings results

We first examined if the affective ratings when reappraising IAPS pictures were different across conditions in the training phase (also see Grecucci et al., 2013a). To calculate the ability to reappraise the stimuli, we calculated the fluctuations of both arousal and valence over the baseline “look” condition (see Figure 1B). We ran paired sample *t*-tests, with participants' subjective ratings separately for both arousal and valence as dependent variables. Both comparisons were all significant, indicating that participants appeared to have learned reappraisal abilities—Valence: down vs. up [*t*_(19)_ = 549, *p* < 0.001]; Arousal: down vs. up [*t*_(19)_ = −419, *p* < 0.001]. Subjects rated their arousal as increasing in the up-regulation and decreasing in the down-regulation, while valence was decreased in the down-regulation (meaning it was less negative), and increased in the up-regulation (more negative).

To understand which were the emotions that might be involved when reappraising the social situation of DG, and to check for confidence when applying the strategies, we analyzed the debriefing questionnaires. Notably, this debriefing exposed subjects to the same kind of stimuli taken from the scanning session, but, added questions to understand (1) the emotions involved, (2) the level of emotional strength and (3) the perceived ability to reappraise. One participant was excluded due to non-completion of the ratings. First, we performed an ANOVA with factors being Fairness (€1 vs. €5) and Type of emotion (anger, sadness, disgust, surprise, happiness). This returned a significant main effect of Fairness [*F*_(1, 19)_ = 15, 000, *p* < 0.001], of Type of emotion [*F*_(4, 76)_ = 7466, *p* < 0.0001], as well as the interaction [*F*_(4, 76)_ = 39, 920, *p* < 0.0001]. Then dependent-sample *t*-tests were performed using subjective ratings for every couple of emotions per time as dependent variables. Results demonstrate that the level of anger significantly differed from most of other emotions [anger-disgust: *t*_(19)_ = 2058, *p* < 0.05; anger-surprise *t*_(19)_ = 2868, *p* < 0.01; anger-happiness: *t*_(19)_ = 6064, *p* < 0.001; anger-sadness: *t*_(19)_ = 296, *p* < 0.05]; disgust differed from happiness [*t*_(19)_ = 4807, *p* < 0.001] but not from surprise [*t*_(19)_ = 1539, *p* = 0.14], and from sadness [*t*_(19)_ = 847, *p* = 0.408]; surprise differed from happiness [*t*_(19)_ = 4578, *p* < 0.001], but not from sadness [*t*_(19)_ = −607, *p* = 0.55]; happiness differed from sadness [*t*_(19)_ = −4188, *p* < 0.001]. However, when correcting for multiple comparisons (Bonferroni, *p* = 0.005) anger did not differ anymore from disgust and from sadness, and surprise did not differ from happiness. Overall, these results indicate that the emotion elicited by the unfair offers in a post scan session identical to the one used in the scanning session, and presumably modulated by the reappraisal strategies when subjects reappraised the DG rounds, was anger followed by sadness and disgust (see Figure 1C). Finally, in a manipulation check, participants were asked to indicate whether they felt their emotions changed according to the strategy adopted (see Figure 1D). Results were computed as deviations from the mean (5 point in a scale from 1 to 9) using dependent-sample *t*-test with subjective ratings for each of two offers as dependent variables. Participant ratings indicate that in the “Down” condition, both fair (5:5) and unfair (1:9) offers were modulated in the predicted direction [respectively, *t*_(1, 20)_ = −2416, *p* < 0.05 and *t*_(1, 20)_ = −3141, *p* < 0.05], while in the “Up” condition only the unfair offer was modulated in the expected direction [*t*_(1, 20)_ = 2234, *p* < 0.05; *t*_(1, 20)_ = 576, *p* > 0.05 for the fair offer]. Please note that these results were also partially presented in a previous study (Grecucci et al., 2013a).

### fMRI results

#### Main effect of strategy

**Figure 2:**
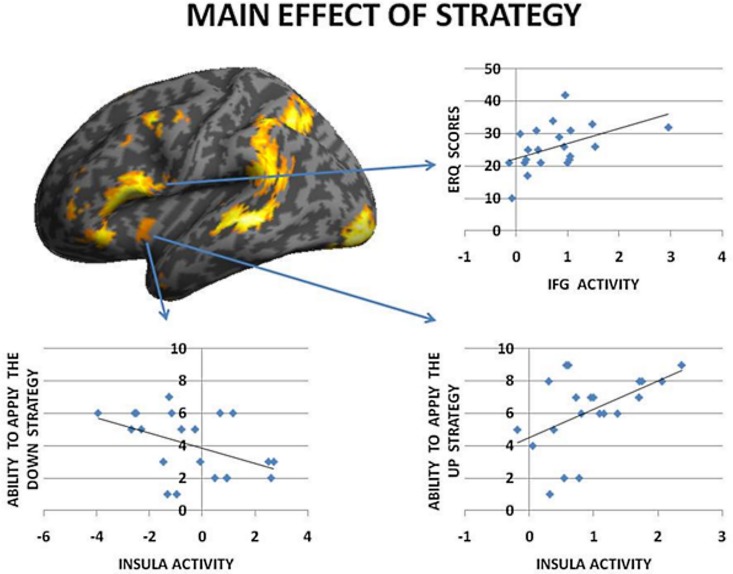
**The main effect of strategy returned significant activations for MFG, IFG, and temporo-parietal activations**. Overall these regions responded more to up- and down-regulation conditions as compared to look, independent of offers. Of these regions the IFG activity correlated with ERQ, and the Insula with the perceived change in emotion as an effect of strategy in the debriefing questionnaire.

To begin with, the main effect of regulation strategy (down + up > look across all trial types) was computed to explore the brain structures involved when applying the strategy reappraisal-mentalizing to the social situation of the DG as compared to the baseline condition of merely observing the offers. This analysis showed activations of, in order of significance, the left middle frontal gyrus, a swathe of temporo-parietal regions bilaterally, the insula bilaterally and the left inferior frontal gyrus. (see Figure 2 and Table 1). In addition, the IFG positively correlated with ERQ measures and insula was positively correlated with the perceived change in emotional response as an effect of up-regulating and negatively when down-regulating (*p* < 0.05), supporting the insula's role in IER.

**Table 1:** **Effect of strategy (all offers UP + DOWN > LOOK)**.

**Anatomical label**	**Voxel**	***H***	***Z***	***p***	**MNI**		
					***x***	***y***	***z***
STG^*^	1119	L	6.01	0.000	−54	−31	4^*^
STG^*^^^^	685	R	5.52	0.000	54	−61	31
MFG	108	L	5.10	0.000	−39	44	7^*^
TP	50	L	5.09	0.000	−57	8	−11
Insula	83	L	4.98	0.000	−45	11	10^*^
TP	44	R	4.69	0.000	57	11	−11
IFG	32	L	4.52	0.000	−57	11	19^*^
Insula	18	R	4.08	0.000	45	11	−11^*^
SFG	17	L	4.21	0.000	−36	14	49

^ inc. TPJ sites,

* p = 0.05 FEW.

#### Separate effects of up- and down-regulation strategies

In order to test for differences between the two regulatory strategies, we separately computed the effects of up- and down-regulation. These contrasts were each computed by comparing to the baseline look condition. Down-regulation strategy involved significant activation of the TPJ bilaterally, the left middle and right superior temporal gyrus and the left inferior frontal gyrus (Table 2A), whereas, the up-regulation strategy revealed the right middle temporal gyrus, the left insula, the right superior temporal gyrus, the left striatum, the left inferior frontal gyrus and the left inferior parietal gyrus (Table 2B). In other words, the way we interpret others' intention (mentalize), affect the activity of brain regions associated with unpleasant emotional reactivity (insula), and with the perception of others (semantic areas in temporal regions).

**Table 2A:** **DOWN regulation (DOWN > LOOK for unfair + midfair)**.

**Anatomical label**	**Voxel**	***H***	***Z***	***p***	**MNI**		
					***x***	***y***	***z***
TPJ	445	R	4.70	0.000	63	−61	16^*^
TPJ	485	L	4.64	0.000	−66	−46	4^*^
pMTG	66	L	4.16	0.000	−57	5	−14
aSTG	49	R	4.13	0.000	54	5	−11
IFG	16	L	3.64	0.000	−48	5	13^*^

**Table 2B:** **UP regulation (UP > LOOK for unfair + midfair)**.

**Anatomical label**	**Voxel**	***H***	***Z***	***p***	**MNI**		
					***x***	***y***	***z***
pMTG^*^	548	R	5.34	0.000	45	−40	−5
^*^TPJ	1078	L	5.27	0.000	−48	−43	22^*^
^*^Insula	682	L	4.84	0.000	−39	8	−2^*^
aSTG	169	R	4.78	0.000	54	8	−11
Striatum	46	L	4.39	0.000	−21	8	4^*^
IFG (9)	39	R	4.22	0.000	−60	11	22^*^
IPL (40)	147	L	4.21	0.000	−45	−43	52
MFG (10)	39	R	3.84	0.000	33	56	4

* p = 0.05 FWE.

#### Regulation effects

Similarly to results of a previous study (Grecucci et al., 2013a), where some activations were reduced when down-regulating and others increased when up-regulating, we expected the effects of the applied strategies to produce varied effects across key brain regions. To test for this hypothesis we computed the contrast down < look < up. The regions modulated by the strategies were, in order of significance, the striatum bilaterally, the posterior cingulate cortex and the insula. Of particular interest for the present paper are the insula for its well-known role in socioeconomic games, the striatum, often modulated in reward experiments (Staudinger et al., 2009), and the posterior cingulate cortex.

**Figure 3:**
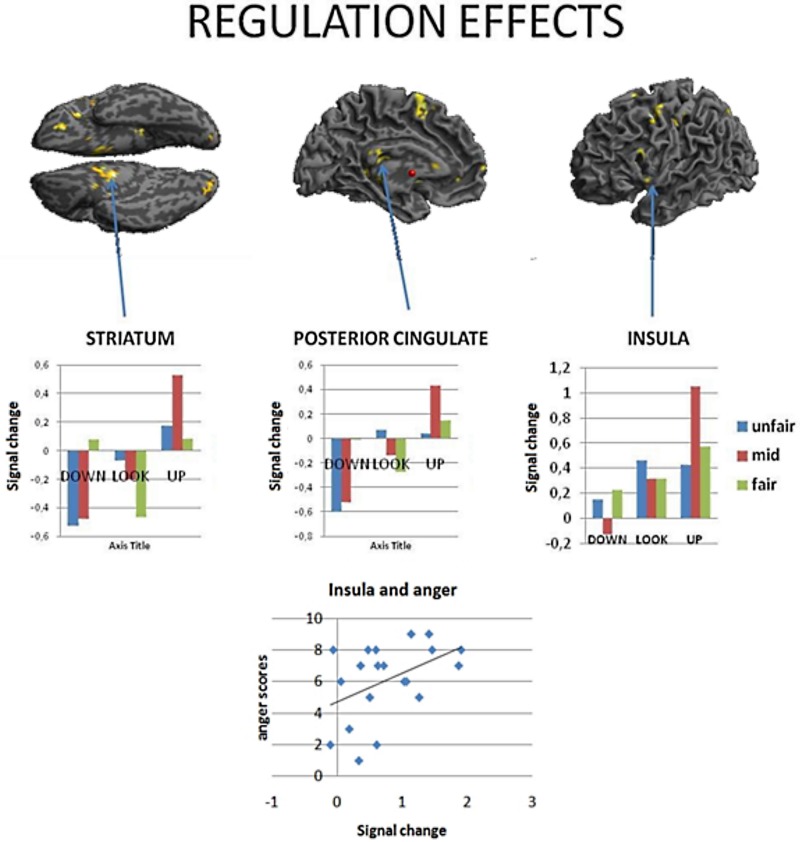
**Regions modulated by reappraisal-mentalizing (down\ look\ up), returned regions showing a linear increase: the striatum, the posterior cingulate and the insula**. Notably, insular activity in this contrast positively correlated with the level of anger when treated selfishly (debriefing).

To better understand the activity patterns of these three regions, we extracted the signal from the voxels from a sphere of 8 mm around the peak of activity using Marsbar toolbox (Brett et al., 2002). As shown in the bar plots, the insula, the cingulate and the striatum were clearly modulated by the strategies, each showing down < look < up behavior (See Figure 3, Table 3). Notably, insula activity was correlated with the level of anger experienced by subjects when receiving a very unfair offer (*p* < 0.05), thus confirming the hypothesis derived from clinical observations that if we perceive in a negative way the intentions of others this will affect our interpersonal emotions and reactions.

**Table 3:** **Effect of regulation DOWN < LOOK < UP for unfair and mid fair**.

**Anatomical label**	**Voxel**	***H***	***Z***	***p***	**MNI**		
					***x***	***y***	***z***
Striatum	388	L	4.08	0.000	−15	5	−19^*^
p.Cingulate	97	R	3.96	0.000	21	−40	25^*^
MiFG	16	L	3.84	0.000	−42	−1	46
VMPFC	17	−	3.81	0.000	0	65	1^*^
paraHippG	43	R	3.61	0.000	42	−37	−2
Striatum	64	R	3.60	0.000	21	5	−2
MeFG	124	L	3.45	0.000	−3	5	58
Insula	30	L	3.20	0.001	−42	11	−2

* p = 0.05 FWE.

#### Interaction of strategy with different types of social behaviors

**Figure 4:**
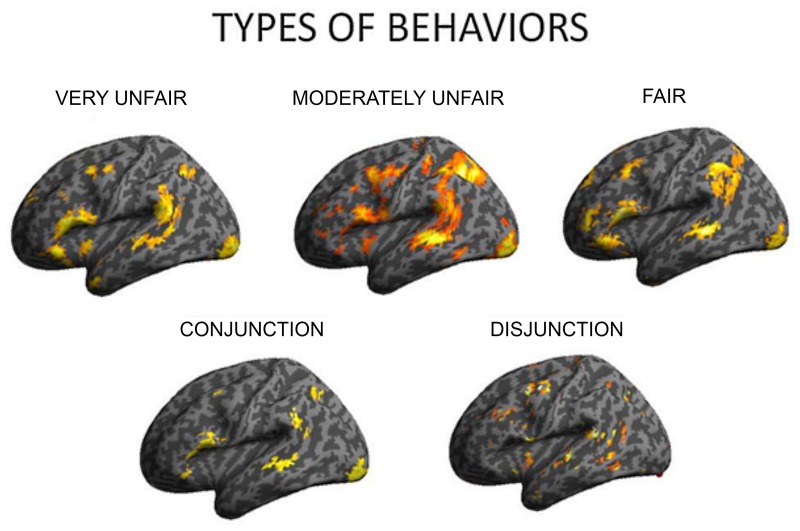
**To understand how different types of behavior affect brain responses, separate analyses were computed for the three levels of offer shown (very unfair, moderately fair, fair)**. Disjunction analyses clarified that insula and other regions differentiated selfish from altruistic behavior.

To examine how the regulation strategies were applied across different types of social behavior observed by the subjects (fair, moderately unfair, very unfair), we computed three separate contrasts for each set of behaviors when regulating the associated emotions (up and down vs. baseline for each of fair, moderately fair and very unfair behaviors). This set of analyses demonstrated several areas commonly activated independent of offer type, but, also some differences. This result was further confirmed when computing conjunction and disjunction analyses for the three contrasts (see Figure 3, as well as Table 4). A conjunction analysis returned the common areas active for all the three types of behaviors, and a disjunction analysis was computed by collapsing between unfair and mid fair (previously exploratory analyses had shown that they were very similar), and contrasting them to the fair condition with exclusive contrast. These analyses returned common areas: the inferior frontal gyrus, the middle temporal and parietal cortices, together with the occipital gyrus (Figure 4, on the bottom left), and also specific areas: the middle frontal gyrus, the TPJ, the insula, and loci on the temporal cortex were only active during moderately fair and very unfair offers (Figure 3 on the bottom right), confirming and extending previous results on this topic (Sanfey et al., 2003; Grecucci et al., 2013a).

**Table 4A:** **Interaction effects (conjunction of all offers)**.

**Anatomical label**	**Voxel**	***H***	***Z***	***p***	**MNI**		
					***x***	***y***	***z***
OG	107	R	4.92	0.000	24	−94	−2
IFG	200	L	4.38	0.000	−45	11	10
OG	188	L	4.28	0.000	−33	−88	−14
MTG	94	L	4.26	0.000	−57	−37	−2
MTG	51	R	3.84	0.000	48	−31	−2

**Table 4B:** **Interaction effects (disjunction between unfair + mid vs. fair offers)**.

**Anatomical label**	**Voxel**	***H***	***Z***	***p***	**MNI**		
					***x***	***y***	***z***
MFG	391	L	5.76	0.000	−42	−1	46	
TPJ	157	L	5.65	0.000	−60	−46	13	
Insula	207	L	5.10	0.000	−48	−1	13	
MTG	57	L	5.01	0.000	−54	−22	−2	

### Dynamic causal modeling

Following the contrast results presented above and based on the previous literature on this topic, we assume that when subjects reappraise their emotions, some regions in the brain are responsible for the implementation of the reappraisal strategy that is they act as “Regulating regions” and some other regions responsible for the emotional appraisal becomes regulated, in other words they can be considered as the “Regulated regions.” Building on this observation we aimed at discovering which region is modulated by the regulating regions that may subserve the regulation of interpersonal emotions. This was done by testing three different models (DCMs) that keep constant the regulating regions (more active regions in the “effect of strategy” contrast, IFG and TPJ), while varying the regulated region (striatum, insula, posterior cingulate). We assume that the model that shows the stronger connection parameter between the regulating regions and the regulated regions is the model that better explain the regulatory effects observed in this experiment.

**Figure 5:**
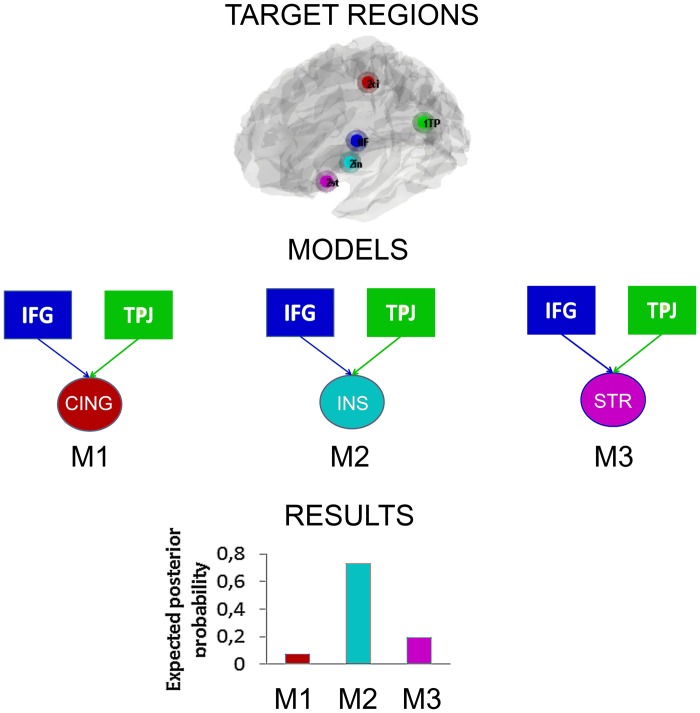
**Dynamic causal models**. In squares the “Regulating regions” and in circles the “Regulated regions.” Three models were tested. Results showed that a model considering the IFG and TPJ acting as modulators and the insula as the regulated regions is the one that better explains the data.

To begin with, we used the same GLM design used for all the contrasts in this paper. Inputs were modeled with the same design matrix of the GLM used in the main analyses. There were three regressors for strategy (down, look, up) multiplied by three regressors for level of fairness (fair, mid fair, unfair), with a total of nine regressors. The contrasts that entered the DCM were the effect of strategy and the effect of regulation (see previous paragraphs), for both unfair and mid fair offers that showed a similar result in previous analyses. Then we selected the meaningful regions to put in the models to test. We extracted time series from spheric volume of interests (VOI) of 8 mm from these five regions using the coordinates derived from the Tables 1, 3, though adjusted for local maxima. We included the two key regions found in the main effect of strategy (down + up > look contrast), namely the IFG (−54, 8, 22) and the TPJ (−54, −46, 28), that reasonably are the structures implementing the reappraisal process and act as modulators. Whereas, from the regulation contrast, the striatum (−21, 14, −17), the posterior cingulate (27, −46, 37) and the insula (−39, 5, 1) were found to be the regions regulated (down < look < up contrast). Previous exploratory analyses reported similar results for separate IFG and TPJ so we assume they are acting in a similar or in concert and thus, we computed three separated DCMs as follow: (1) Regulating regions: IFG + TPJ, Regulated: Striatum, (2) Regulating regions: IFG + TPJ, Regulated: insula, (3) Regulating regions: IFG + TPJ, Regulated: posterior cingulate, in order to test the idea of which region is modulated by IFG and TPJ. Moreover, we tried different combinations of connections (feedforward and backforward), tested for both up and down regulation conditions. However, results derived from different types of connectivity and for both regulations, led to similar results. For the matter of simplicity, we reported only results derived from the up-regulation for unfair and mid fair offers, and feedforward connections (hierarchically organized from IFG and TPJ to each of the three target regions) analyses. The three models were estimated with a Bayesian model comparison. Results reported in Figure 5, show clearly a preference for model 2 (Regulating regions: IFG + TPJ, Regulated region: insula).

## Discussion

In the present study we show the neural correlates of IER, that is, regulatory strategies applied to socially evoked emotions. As detailed below, this study extends previous studies on this topic, exploring for the first time whether cognitive regulation strategies modulate brain responses of social emotions (e.g., affective response to being treated well or poorly by another). Previous findings on the neural substrates of cognitive reappraisal were replicated, while also extended to uncover brain structures more generally involved in both mentalizing and interpreting other's intentions in a more or less negative way.

### Brain correlates of interpersonal emotion regulation

During the acquisition of reappraisal strategies subjects were capable of successfully modulating their perception of the valence and arousal levels of training stimuli. Unpleasant pictures taken from the IAPS database were rated as more arousing and more negative in the up-regulation condition as compared to baseline, and conversely less arousing and less negative in the down-regulation, again compared with the baseline condition. Further assessment of strategy application revealed significant modulation of the ability to down- and up-regulate on command. This allowed us to address four primary questions here. Firstly, we sought to confirm previous studies on ER that have outlined a role for inferior frontal gyrus in implementing reappraisal strategies (see Wager et al., 2009; Ochsner et al., 2012, for reviews). We confirmed this point, showing clear activation of the IFG when asking which brain regions were generally responsible for reappraising the intentions of others. This finding further extends the role of this region in reappraising, by demonstrating its involvement in interpreting another emotional state, this time anger when treated unfairly in a social interactive context. This region has also been observed in a previous study about socioeconomic decision-making using a different task (see Grecucci et al., 2013a).

Using for the first time a social interactive task independent of a decision-making situation allows for exploration in more detail of brain regions associated with different kinds of behavior. This manipulation showed strong involvement of social and mentalizing related regions. The temporo-parietal areas, as well as the medial prefrontal cortex including the paracingulate cortex, have been implicated in mentalizing (Frith et al., 1991; Frith and Frith, 2003) and intention-detection, and may be particularly important here when considering that reappraisal strategies specifically lead participants to reinterpret the intentions of their opponents, as assessed by self-report measurements taken after scanning. Making sense of social interactions requires inferring intentions, beliefs, and desires, that is attributing mental states (i.e., mentalizing; see Frith et al., 1991). This was exactly what players were doing when applying the reappraisal strategies, and other recent studies have pointed out that mentalizing abilities are involved when making socially valued decisions (Evans et al., 2011). In sum, this study can extend actual model of ER such as the MCCE of Ochsner et al. (2012), suggesting that TPJ should be included in the list of regions acting as modulators, in addition to the previously cited dlPFC, ACC, vlPFC, and dmPFC.

Another goal of the present experiment was to study brain responses when facing different kinds of social behaviors from another, from a fair interaction based on equity to increasingly unfair scenarios based on inequity and selfishness. Insula was found to be the key region in differentiating the selfishness of another's social behavior.

### Mentalizing interpersonal emotions

Another finding of this paper was the detection of areas potentially responsible for appraising and reappraising social emotions. The regions implicated here were the striatum, the posterior cingulate cortex and the insula. Interestingly, the striatum has been involved not only in primary or secondary rewards, but also to more abstract, social rewards (van den Bos et al., 2013). One hypothesis is that when subjects engage in social interactions such as the one induced by the DG, the associated social reward value is changed according to the success of this interaction. Therefore, the regulation strategies may affect this region's response in such a way as to adjust the social value when treated unfairly, depending on the reappraisal strategy used. Importantly, when mentalizing in a negative way, activity in the striatum is increased. This mechanism may serve to evaluate and “label” the unfair partner and adjust future interactions with the same partner. Indeed, it was recently proposed that striatum plays a role in reputation formation, another aspect of regulating our reactions when interacting with others (Engelmann and Hein, 2013).

Another region, modulated by the strategy was the posterior cingulate. This is in accordance with previous findings on perceiving negative emotions, especially anger (Murphy et al., 2003), and on regulating emotions induced by simple visual stimuli (Ochsner et al., 2004a,b; Goldin et al., 2008), thus extending the role of these areas into regulating more complex socio-economic emotions.

Last but not least, the insula has been previously reported in the context of the UG, and shown to be involved in responses to unfair offers in particular (Sanfey et al., 2003), and also when modulating the associated decision to reject them (Grecucci et al., 2013a). Consistent with previous studies (Pillutla and Murnighan, 1996; Xiao and Houser, 2005), post-scanning debriefing indicated that anger was the primary emotion elicited by a selfish interactions. Interestingly, neural evidence of the involvement of the insula in the emotion of anger has recently been shown (Denson and Nandy, 2009). One difference with the previous study mentioned above is that in Grecucci et al. (2013a) two regions of the insula where found to be active, one more anterior and one more posterior. In the present study only the anterior insula was modulated by the strategies. Activation of bilateral anterior insula to unfair behavior when interacting with a partner is particularly interesting in light of this region's association with negative emotional states (Sanfey et al., 2003). This region has also been implicated in studies of emotion, in particular involvement in the evaluation and representation of specific negative emotional states (Calder et al., 2001). With respect to emotion-processing systems, it has been hypothesized that reappraisal would modulate the processes involved in evaluating a stimulus as affectively significant (Goldin et al., 2008). Reappraisal effectively down-regulates emotion related neural responses that together modulate ongoing emotion experience in emotion-appraisal brain systems, including the amygdala, subgenual ACC, ventromedial PFC, and insula (Ochsner et al., 2004a,b; Ochsner and Gross, 2005; Eippert et al., 2007; Grecucci et al., 2013a). If the activation in the anterior insula is a reflection of the responders' negative emotional response to an unfair offer, we might expect activity in this region to correlate with the degree to which subjects apply the reappraisal strategies, which is indeed what was found. The better subjects are at down-regulating their emotions, the less the insula is active, whereas, the better subjects are at up-regulating their emotions the more this regions is active. The role of this region in reappraising social emotions was also confirmed by further tests using DCM as a way to explore the network implied in effective regulation. These analyses showed that a circuit including IFG and TPJ acting as modulatory structures and the insula as the region modulated, is responsible for the regulation of socially induced emotions. One hypothesis is that the insula represents the mean by which cognitive strategies can modulate the arousal associated with emotions (Grecucci et al., 2013a). Indeed, other regions found to be modulated by the strategies in the GLM analysis (cingulate cortex and striatum) were not found to be modulated by IFG and TPJ when considering DCM. It is typically assumed that the beneficial effects of reappraisal are accomplished via interactions between PFC regions and subcortical networks related to emotional responding (Beauregard et al., 2001; Ochsner et al., 2004a,b; Kalisch et al., 2005; Phan et al., 2005; Urry et al., 2006; Eippert et al., 2007; Kim and Hamann, 2007; van Reekum et al., 2007; Goldin et al., 2008; Wager et al., 2009). In particular, Wager and collaborators showed with pathway-mapping analysis that a circuit including the ventro-lateral prefrontal cortex (close to the IFG of the present study) and target emotional regions (nucleus accumbens and amygdala) are responsible for regulation strategies.

### Clinical implications

The present study has also relevance for understanding some clinical phenomena such as paranoid thinking and interpersonal skills deficits. Psychotherapists as well as psychiatrists, know that the way we interpret the intentions of others can deeply affect emotional reactions (Allen and Fonagy, 2006; Clarkin et al., 2006), and interpersonal behavior (Linehan, 1993). The more we perceive the intentions of others as malevolent, the more negative emotions we feel, and the more we respond to others in a bad way. In the present study we studied what happens when subjects mentalize in a negative way the intentions of others (up regulation condition). We found that this thinking strategy (implemented in the IFG and TPJ) increases activity in brain structures responsible for emotional reactions (such as the insula and the striatum), and areas associated with the perception of others' mind (middle and superior temporal gyrus?). Notably, when mentalizing in a negative way, insula's activity correlates with the level of anger when treated selfishly. Overall, these data confirm clinical previous observations stating that interpreting others' intentions in a negative way, increases inappropriate interpersonal emotional reactions by affecting the perception of others.

### Limitations and future directions

Lastly, we acknowledge some of the limitations that characterize the present study. First, the lack of internal emotional rating during the scanner limits the connection of the neural results with the corresponding subjective level. However, it should be pointed out that there is supporting evidence that the manipulation was affecting the subjective-behavioral level, as the same subjects also played the Ultimatum Game in which we showed strong behavioral modulation of subjects' decisions when applying the strategies (see Grecucci et al., 2013a). Future studies will have to assess at a more behavioral-subjective level the effect of reappraisal strategies in regulating social emotions (Grecucci et al., 2013b). Moreover, in the present study we did not include a measure to assess the quality of interpersonal transaction, though a previous study used the percentage of rejection rates of the partners' proposals (Grecucci et al., 2013a). Future studies may include subjective or behavioral indexes in order to have a quantitative measure of this. Last but not least, the DCM results should be considered as exploratory and more complex models may be addressed in future research.

## Conclusion

Previous studies have reported the effect of ER strategies in the self, however, the effect of regulation on socially driven emotions was still unclear. Here we show for the first time that IER strategies can strongly affect neural responses when experiencing socially driven emotions, thus extending actual models of ER.

### Conflict of interest statement

The authors declare that the research was conducted in the absence of any commercial or financial relationships that could be construed as a potential conflict of interest.
